# *QuickStats:* Percentage* of Adults Aged 18–26 Years Who Ever Received a Human Papillomavirus Vaccine,^†^ by Race and Hispanic Origin^§^ and Sex — National Health Interview Survey, United States, 2019^¶^

**DOI:** 10.15585/mmwr.mm7021a5

**Published:** 2021-05-28

**Figure:**
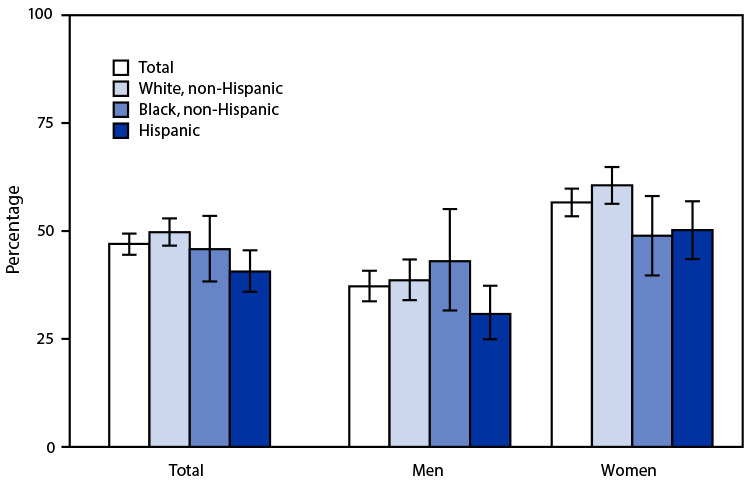
Overall, in 2019, 47.0% of adults aged 18–26 years had ever received an HPV vaccination. Non-Hispanic White adults (49.7%) were more likely than Hispanic adults (40.6%) to have ever received an HPV vaccination; differences between non-Hispanic Black adults (45.8%) and the other two groups were not statistically significant. Overall, women were more likely than men to have been vaccinated (56.6% versus 37.2%), and this pattern was seen for non-Hispanic White women and men (60.6% versus 38.6%) and for Hispanic women and men (50.2% versus 30.8%). However, the difference between non-Hispanic Black women and men (48.9% versus 43.0%) was not statistically significant.

**Source:** National Center for Health Statistics, National Health Interview Survey, 2019. https://www.cdc.gov/nchs/nhis.htm

